# Wired to be connected? Links between mobile technology engagement, intertemporal preference and frontostriatal white matter connectivity

**DOI:** 10.1093/scan/nsz024

**Published:** 2019-04-25

**Authors:** Henry H Wilmer, William H Hampton, Thomas M Olino, Ingrid R Olson, Jason M Chein

**Affiliations:** 1 Department of Psychology, College of Liberal Arts, Temple University, Philadelphia, Pennsylvania, USA; 2 Decision Neuroscience, College of Liberal Arts, Temple University, Philadelphia, Pennsylvania, USA

**Keywords:** white matter, smartphones, mobile technology, delay discounting, impulsivity, reward, diffusion imaging, connectivity

## Abstract

Youth around the world are increasingly dependent on social media and mobile smartphones. This phenomenon has generated considerable speculation regarding the impacts of extensive technology engagement on cognitive development and how these habits might be ‘rewiring’ the brains of those growing up in a heavily digital era. In an initial study conducted with healthy young adults, we utilized behavioral and self-report measures to demonstrate associations between smartphone usage habits (assessed both subjectively and objectively) and individual differences in intertemporal preference and reward sensitivity. In a follow-up neuroimaging study, we used probabilistic tractography of diffusion-weighted images to determine how these individual difference characteristics might relate to variation in white matter connectivity, focusing on two dissociable pathways—one connecting the ventral striatum (vSTR) with the ventromedial prefrontal cortex (vmPFC) and the other connecting the vSTR with the dorsolateral prefrontal cortex (dlPFC). Regression analyses revealed opposing patterns of association, with stronger vSTR–vmPFC connectivity corresponding to increased mobile technology engagement but stronger vSTR–dlPFC connectivity corresponding to decreased engagement. Taken together, the results of these two studies provide important foundational evidence for both neural and cognitive factors that can be linked to how individuals engage with mobile technology.

## Introduction

Over the past two decades, personal electronic devices have become increasingly mobile and powerful, a facet of what is popularly known as the ‘smartphone revolution’. Recent estimates suggest that nearly nearly 80% of Americans now own a smartphone (Pew Research Center Mobile Fact Sheet, 2018), some of whom average upwards of 300 min per day on these devices (Andrews *et al.*, 2015). The near omnipresence of the ‘digital world in our pockets’, and the ease with which we can acquire information and desired rewards by engaging with it, may influence foundational processes of cognition and affect in both beneficial and harmful ways. While many people find productive and fruitful ways to embed mobile technology into their daily routines, many also report feeling ‘addicted’ to their mobile devices (Martinotti *et al.*, 2011). Difficulty disengaging from one’s devices can have a variety of consequences, from relatively benign, e.g. appearing rude to a friend by not maintaining eye contact during a meal, to life-threatening, e.g. distracted driving, which in 2017 caused 2935 crashes and 3166 fatalities (National Highway Traffic Safety Administration, 2019). A now rapidly growing scientific literature seeks to inform our understanding of the individual differences factors that might predict variation in digital media use behaviors and to explore how these factors might interact with the potential benefits and harms that such behaviors could evince (Wilmer *et al.*, 2017).

### A link between ‘impatience’ and smartphone/social media use habits

Among the most commonly repeated concerns regarding societal smartphone and social media dependence is that such behaviors may engender in users an especially acute sense of impatience and, along with it, a distaste for situations in which one must wait out or plan for longer-term outcomes. A typical manifestation of this assertion focuses on ‘today’s adolescents’, who, having been raised in an environment suffused in digital technology, have cultivated an especially strong need for instant gratification and a diminished ability to plan effectively for the future (Muther, 2013).

Such claims regarding the potential negative impacts of emerging technologies are nothing new and quite often turn out to be unfounded. Moreover, causal claims of this type call for evidence that goes beyond the observational and correlational studies that typify the early scientific literature in this domain. Still, correlational findings can be instructive in guiding our causal hypotheses, and the absence of predicted correlations can serve as key evidence against causal claims. Accordingly, mounting correlational evidence suggesting that increased mobile technology use is in fact positively associated with a heightened preference for immediate rewards (Atchley and Warden, 2012; Hayashi *et al.*, 2015; Wilmer and Chein, 2016; Hadar *et al.*, 2017; Tang *et al.*, 2017) provides an important starting point for any argument regarding the impacts of such behaviors.

### Associations between mobile technology habits and the brain

Along with a growing literature investigating the links between technology usage and cognitive/affective functioning (Wilmer *et al.*, 2017; Marty-Dugas *et al.*, 2018), there has emerged a body of work exploring the brain mechanisms by which these behaviors may be connected (Mills, 2014; Meshi *et al.*, 2015; Crone and Konijn, 2018). Much of this work examines mobile technology engagement (MTE) by way of social media use. Early functional magnetic resonance imaging (fMRI) research on the specific neural correlates of social media behavior highlighted the role of the ventral striatum (vSTR), including the nucleus accumbens and immediately neighboring areas, as a key locus of activity. Meshi *et al.* (2013), for instance, showed that social media engagement (indexed by Facebook usage) was related to the degree of accumbens recruitment in response to social feedback that was perceived to influence participants’ social reputation (a proxy for ‘likes’ encountered on social media), while the accumbens response to non-social monetary rewards was not related to social media behavior. In further work, Sherman *et al.* (2016) observed greater activation of the vSTR (and ventral tegmental area) in response to shared images that were rated as more popular (based on number of experimentally manipulated ‘likes’ received) than those receiving weaker social endorsement. These functional observations are complemented by structural evidence indicating that the total gray matter volume of striatal regions is correlated with social media habits (daily smartphone checking of social media in Montag *et al.*, 2017; excess social media use in He *et al.*, 2017, which also found a link between amygdala volume and social media). One further relevant study (Turel *et al.*, 2018) similarly explored potential links between gray matter structure, social media habits (Facebook usage) and delay discounting (DD) behavior. While that study failed to observe correlations between social media use and gray matter volume in striatal regions, a link between social media use and gray matter morphology in a region contributing to the broader reward processing network—the posterior insula—was observed, and this relationship was significantly mediated by DD behavior.

Other work, meanwhile, points to a role for regions implicated in executive/impulse control in relation to smartphone and social media usage behaviors. Loh and Kanai (2014), for example, found reduced gray matter in the anterior cingulate cortex of frequent media multitaskers (e.g. combining smartphone use with other electronic media such as video game play or TV watching), indicating that this habit may have a direct impact on the structural properties of an important locus of inhibitory control in the brain. Subsequently, Moisala *et al.* (2016) demonstrated that heavy media multitaskers also exhibit increased activity in the right prefrontal cortex (PFC) during a cognitively demanding task, a finding the authors interpreted to reflect the greater difficulty these individuals have with recruiting executive control resources. This interpretation receives corroboration from a further recent study finding that heavy smartphone users show a weaker evoked potential (measured through electroencephalography) in response to brief transcranial magnetic stimulation of the right PFC (Hadar *et al.*, 2017), which may indicate decreased resting-state glutamatergic excitability in this region among heavy smartphone users. Findings linking digital media behaviors to the brain’s self-regulatory control structures suggest that variation in individuals’ ability to exert goal-directed control over behavioral impulses might also act as a key psychological pathway linking mobile technology habits to the brain. And again, early correlational work showing significant associations between smartphone and social media use habits with measures of impulse control (Wilmer and Chein, 2016) provides some important corroborative evidence.

A broader literature based on the use of diffusion weighted imaging (DWI) to characterize white matter pathways in the brain also provides useful guidance with respect to the connective tracts that are most likely to be implicated in smartphone use and its association with cognition and affect. Prior work suggests, for instance, that structural white matter connectivity between the striatum and the frontal lobes is especially predictive of individual differences in several reward and decision-processing tasks (Liston *et al.*, 2006; Cohen *et al.*, 2009; Samanez-Larkin *et al.*, 2012). With specific regard to intertemporal preference (i.e. DD), Peper *et al.* (2013) showed that greater structural integrity in the connections between the striatum (across its full dorsal to ventral extent) and PFC was associated with decreased discounting of delayed rewards. Providing some further anatomical granularity, van den Bos *et al.* (2014) showed that greater connectivity specifically between the mid-striatum and the dorsolateral PFC (dlPFC) was associated with less discounting of delayed rewards. Meanwhile, based on a common structural distinction between ventral and dorsal subregions of the striatum, Hampton *et al.* (2017) hypothesized that these loci may also be unique in terms of their white matter connectivity to other brain regions, and the associations between brain connectivity and immediate gratification seeking. As anticipated, Hampton *et al.* (2017) found dissociable patterns wherein greater connectivity between the left vSTR and ventromedial PFC (vmPFC) uniquely predicted steeper discounting on a DD task, whereas connectivity between the left dorsal striatum (dSTR) and vmPFC was not associated with discounting performance. To date, we are aware of two studies that have examined structural brain connectivity in relation to mobile technology habits (Hu *et al.*, 2017; He *et al.*, 2018). In both studies, individuals with severe smartphone dependence were found to exhibit white matter abnormalities (relative to controls). In Hu *et al.* (2017) these abnormalities were observed across several midline pathways and the superior longitudinal fasciculus, while in He *et al.* (2018) these abnormalities were observed in the corpus callosum. As of yet, no work has simultaneously explored associations between white matter connectivity and intertemporal preference with respect to individual differences in mobile technology habits that fall in the ‘healthy’ range (i.e. for a normative sample without known technology dependence concerns).

### Measuring smartphone usage behavior and aims for the present study

One shortcoming of much prior research on smartphone usage behaviors is that studies have depended almost entirely upon coarse-grained and subjective self-report measures (e.g. Ophir *et al.*, 2009; Wilmer and Chein, 2016) that may not generalize well to actual usage behavior (Andrews *et al.*, 2015). This dependence on self-report indices arose due to the fact that, until quite recently, there was no reliable and non-intrusive way to objectively monitor specific facets of smartphone behavior. However, recent changes in the operating systems that undergird smartphone functioning, and to the apps that run on these platforms, have opened the door to alternative methods for collecting usage data (Rich *et al.*, 2015; Byrne *et al.*, 2017). In the present study, we capitalized on the fact that the operating system that is now standard on all modern Apple iPhones (iOS 8 or above) includes a function that automatically tracks the time that a user spends with each individual application showing on the front of the phone’s display, providing an objective measure of specific smartphone usage behavior. Accordingly, in Study 1, we compare patterns of usage behavior measured subjectively (self-report) and objectively (based on battery usage reports) in relation to individual differences in impulse control, reward sensitivity and intertemporal preference. By using dependent measures that partially overlapped with those deployed in a prior study of smartphone habits (Wilmer and Chein, 2016), the current work also afforded an important opportunity to explore the replicability of our earlier findings, a pursuit strongly encouraged by recent concerns over replicability of scientific research (Open Science Collaboration, 2015; though, see Anderson *et al.*, 2016 and Gilbert *et al.*, 2016a, 2016b).

In Study 2, we utilize probabilistic tractography of DWI data to shed additional light on the specific neural pathways that undergird the relationships observed in Study 1 and to provide convergent evidence regarding the involvement of particular processes in the association between intertemporal choice and mobile technology habits. Specifically, we sought to first isolate discrete frontostriatal white matter tracts (following the methods of Hampton *et al.*, 2017) and to then examine whether individual variability in connectivity within these parcellated pathways was associated with MTE, as well as with the constructs of intertemporal preference, impulse control and reward responsiveness.

## Study 1

### Methods

#### Participants

One hundred and ten Temple University students participated in Study 1, in exchange for course credit. Four participants were removed from analyses for not properly following task instructions, leaving a final sample of 106 (69.8% female; age *M* = 20.1; s.d. = 2.48). The sample was racially diverse (67.9% self-identified as Caucasian or white, 13.2% as African–American or black, 10.4% as Asian, 5.7% as more than one race and 2.8% declined to respond). All participants were at least 18 years of age and were fluent in written and spoken English. All procedures were approved by the Institutional Review Board at Temple University.

#### Procedure

Each participant was orientated to the general purpose of the study and was asked to sign a consent form that explained the protocol in detail. The experimenter then asked the participant to hand over his or her phone, which was held outside of the testing room in silent mode, such that it would not cause a distraction (Ward *et al.*, 2017). The study commenced with participants completing an initial set of questionnaires, which included the ‘MTE scale’, ‘media multiuse questionnaire’, ‘mobile phone problematic usage scale’ (MPPUS), ‘Zuckerman impulsive sensation seeking scale’, ‘Barratt impulsiveness scale’ and ‘Behavioral inhibition system/behavioral approach system (BAS) questionnaire’, each detailed below. Participants then completed two computer-based behavioral tasks: go/no-go (a measure of impulse control) and DD (a measure of one’s inclination toward more immediate gratification). Finally, actual smartphone usage (ASU) information was collected for a subgroup of participants, as described below.

#### Measures

##### MTE scale

A primary measure of participants’ self-reported engagement with mobile technology was obtained using the MTE scale (Wilmer and Chein, 2016). By assessing self-reported behaviors regarding different facets of mobile technology, the index characterizes broad smartphone engagement patterns. In subsections, the scale assesses phone-based social media use, frequency of public status updating and phone-checking behavior. Responses to each question are given on a Likert-style scale, and aggregate responses are combined based on *z*-scores in each subsection to form a single MTE score for each participant. The overall MTE measure has previously yielded acceptable internal reliability (*α* = 0.65; Wilmer and Chein, 2016) and in the present sample yielded similar reliability (*α* = 0.68).

##### Media multiuse questionnaire

We also gathered information regarding the participants’ technology multitasking habits using a revised form of the media multiuse questionnaire (Ophir *et al.*, 2009). This instrument provides an estimate of the amount of time one spends multitasking with various forms of media, which is aggregated as the ‘media multitasking index’ (MMI). In its original form, participants were asked to estimate the total number of hours they spent engaging in 12 different forms of media (e.g. watching television, playing video games, talking on the phone, instant messaging, etc.) and to specify, across a series of pages (one for each media type), the degree to which they use each media technology concurrently with each of the other media formats (i.e. engage in media multitasking). As in Wilmer and Chein (2016), we used a matrix-style version of the media multiuse questionnaire which allowed participants to detail their media multitasking habits on a single computerized form, rather than across a series of repeated forms pertaining to each media type. The MMI score is an aggregated score based on the sum total of multitasking habits (specific calculation is described in Ophir *et al.*, 2009).

##### Mobile phone problematic usage scale

As a third self-report measure of mobile phone usage, we used the MPPUS (Bianchi and Phillips, 2005). This scale seeks to assess excessive or problematic mobile phone usage by way of a 27-item questionnaire. All questions are responded to on a 10-point Likert scale from 1 (not at all true) to 10 (very true). Aggregate scores on this overall questionnaire can range from 27 to 270. Bianchi and Phillips demonstrated a high level of internal consistency for this scale (*α* = 0.93), and the scale produced a lower but still good level of internal consistency for the current sample (*α* = 0.82).

##### Actual smartphone usage

As a way to cross-validate self-reported smartphone use patterns, we obtained an objective index of ASU by using a native Apple iPhone (iOS 8 or above) feature (built into the battery usage interface), which automatically tabulates the amount of time that a user spends engaging with specific phone applications. At the time of data collection, non-Apple mobile phones did not natively record such information, and thus only iPhone data was collected. With the owner’s permission, the investigators collected screenshots of the phone’s complete battery usage report and coded the detailed individual app data records contained in those screenshots into an aggregated data report. Full iPhone usage data were tallied to index the overall amount of time each participant spent on their phone, and within individual app categories, during the 7 day period prior to arrival for the assessment session. Since only a portion of participants were iPhone owners with suitably updated operating system versions (and with a full 7 day recording period prior to the assessment), objective usage data were obtained from only a subsample (*n* = 56) of the overall participant group.

##### Intertemporal preference

We assessed individual differences in the tendency to delay gratification in favor of larger, later, rewards via a DD task used previously in similar samples (O’Brien *et al.*, 2011; Wilmer and Chein, 2016). In the task, participants were asked to make hypothetical choices between a smaller sum of money offered now *vs* a larger sum of money (always $1000) offered at six different delays, ranging from 1 day to 1 year. The smaller sum of money offered was varied systematically, until the participant reached an indifference point—the value at which the subjective value of the smaller immediate offer matched the subjective value of the larger ($1000) delayed offer (Ohmura *et al.*, 2006). Participants completed 10 trials at each delay interval. Based on prior findings (O’Brien *et al.*, 2011; Weigard *et al.*, 2014; Wilmer and Chein, 2016), responses to the longer delays (6 months and 1 year) were expected to have the greatest individual subject variance, and we found this to again be the case in the present sample (e.g. s.d. for long delays = 275; s.d. for short delays = 164). Therefore, as we had done in previous work (Weigard *et al.*, 2014; Wilmer and Chein, 2016), we averaged the subject’s empirically ascertained indifference point (the point at which the subjective value of the immediate offer matched the subjective value of a delayed offer of $1000) for the three longest delays in order to create a composite measure of DD behavior. We additionally calculated each individual’s discount rate (*k*), and, as is typically done, *k* values were log transformed to address non-normality.

##### Reward sensitivity

Two instruments were used to create a reward sensitivity construct: a subset of questions from Zuckerman’s revised impulsive sensation seeking scale and a subscale of the ‘BIS/BAS’ questionnaire. The impulsive sensation seeking measure (Zuckerman *et al.*, 1993) is a 19-item self-report questionnaire that intentionally conflates impulsive and sensation seeking behaviors in order to broadly characterize these personality traits. Following Steinberg (2010), and replicating our approach in Wilmer and Chein (2016), we used only a subset of six items from the updated Zuckerman scale that most purely related to the sensation seeking construct. This subset of items has been shown to exhibit good internal consistency (*α* = 0.70; Steinberg, 2010) and in the present sample exhibited slightly greater consistency (*α* = 0.80). These items were answered as either true (coded 1) or false (coded 0), and item scores were averaged to create a mean sensation seeking score. As an additional measure of reward sensitivity, we used the reward responsiveness subscale of the BAS component of the ‘BIS/BAS’ scales (Carver and White, 1994). This subscale has been shown to have good internal consistency (*α* = 0.73; Carver and White, 1994) and yielded the same degree of consistency in this sample (*α* = 0.73). As in Wilmer and Chein (2016), a composite reward sensitivity score was created for each subject by calculating the subject’s averaged *z*-score across the sensation-seeking and reward-responsiveness indices.

##### Impulse control

We created a composite impulse control index by averaging standardized scores from two measures, the Barratt impulsiveness scale and false alarm rate on a behaviorally assessed go/no-go task. The Barratt impulsiveness scale is a widely used self-report measure of impulsivity (Patton *et al.*, 1995). Again following Steinberg (2010), we selected 18 items from the full 30-item questionnaire having specificity with respect to impulsive behavior. Each item was answered on a 4-point scale (‘rarely/never’, ‘occasionally’, ‘often’ and ‘almost always’) and scores were averaged, with higher scores indicative of greater impulsivity. This subset of questions has been found to have good internal consistency (*α* = 0.73; Steinberg, 2010) and produced a similar degree of consistency in the present sample (*α* = 0.75).

The go/no-go task used in the current study involved the rapid presentation of a series of go (‘*x*’) and no-go (‘*k*’) stimuli. Participants were instructed to give a button press response following each ‘*x*’ but to withhold responding whenever they saw a ‘*k*’ stimulus. The stimuli were presented for 250 ms each, followed by an unpredictable inter-trial interval (ITI) ranging between 750 and 1750 ms. In total, 333 stimuli were presented, of which 50 were no-go trials (*k*’s). The no-go trials were pseudo-randomly interspersed into the series such that a no-go trial was equally often preceded by 1–10 prior go trials (five occurrences of each). The entire task lasted just over 8.5 min. Normalized scores from both the Barratt impulsiveness scale and go/no-go measure were inverted so as to reflect ‘impulse control’ rather than impulsivity (i.e. a higher score on the construct indicated a stronger tendency to control impulsive responses).

## Results and discussion

Descriptive statistics for all self-report and behavioral variables are provided in Table 1, and bivariate Pearson correlations are summarized in Table 2 (Supplementary Table S1 provides the complete correlation table for all study measures). The three media use questionnaires exhibited positive inter-correlations, with a significant correlation observed for MTE and MMI (*r* = 0.388; *P* < 0.001) and also for MTE and MPPUS (*r* = 0.340; *P* < 0.001). The relationship between the MMI and the MPPUS was trending (*r* = 0.182; *P* = 0.067) but did not reach significance in this sample. Notably, ASU, as measured objectively by total time spent on the smartphone over the prior seven days, also correlated significantly with MTE scores (*r* = 0.332; *P* = 0.012), though it did not correlate with either the MMI or MPPUS scores (*P* > 0.50).

**Table 1:** Descriptive statistics for measures in Study 1

	**Mean**	**s.d.**	**Minimum**	**Maximum**
MTE scale				
Social media use	16.21	4.98	4	27
Public updating	2.33	1.2	1	7
Checking	7.46	2.09	3	14
MMI	3.56	1.5	0.75	7.22
MPPUS	86.81	5.19	31	175
ASU (minutes)	1448.53	524.46	598	2852
Intertemporal preference				
DD average indifference point ($)	572.57	275.98	1	999
DD LogK	−5.85	1.98	−12.47	−1.16
Impulse control				
Go/no-go false alarms	19.32	8.24	1	40
Barratt impulsivity (reverse scored)	1.97	0.36	1.39	3.33
Reward sensitivity				
Zuckerman sensation seeking	0.57	0.31	0	1
BAS reward	16.6	1.89	12	20

DD = Delay Discounting. BAS = Behavioral Approach System.

**Table 2:** Bivariate Pearson correlations for measures in Study 1

	**MTE**	**MMI**	**MPPUS**	**ASU**	**DD**	**IC**	**RS**	**Age**	
**MTE**		0.39^*^	0.34^**^	0.33^*^	−0.19^*^	−0.05	0.36^*^	−0.11	
**MMI**			0.18^†^	0.03	−0.04	−0.21^*^	0.11	0.01	
**MPPUS**				0.06	−0.03	−0.13	0.20^*^	−0.22^*^	
**ASU**					−0.29^*^	0.07	0.06	−0.17	
**DD**						0.11	0.03	−0.03	
**Impulse control**							−0.13	−0.003	
**Reward sensitvity**								−0.12	
**Age**									

Delay Discounting indexed by average indifferent point at long delays on the delay discounting task. Impulse Control signifies composite impulse scores; Reward Sensitivity signifies composite reward scores; MTE = Mobile Technology Engagement, MMI = Media-Multitasking Index, MPPUS = Mobile Phone Problematic Usage Scale, ASU = Actual Smartphone Usage. ^†^*P* < 0.10; ^*^P < 0.05; ^**^P < 0.01.

The present observation of a significant positive correlation between MTE and the MMI replicates a key finding of Wilmer and Chein (2016), which found a similar association between these two measures. Results from Study 1 also extend that finding by showing a significant correlational relationship between MTE and MPPUS. Moreover, these data corroborate the external validity of at least the MTE self-report measure by showing that participants’ responses to that scale were significantly correlated with the total amount of time they actually spent on their phones, as assessed by an objective and non-intrusive measure of actual usage. Accordingly, the results suggest that the MTE successfully condenses aspects of usage that are assessed both by other self-report scales and by actual use patterns.

We next examined correlations between the participants’ MTE scores, ASU and main psychological constructs of the study. Consistent in both direction and magnitude with our previous finding (Wilmer and Chein, 2016), we observed a significant positive correlation between MTE scores and average mean indifference points for long delays in the DD task (*r* = −0.194; *P* = 0.047), though the correlation between MTE and participants’ discounting rate (log(k)) did not approach significance (*r* = 0.12; *P* = 0.22). An association between smartphone usage and DD was, however, also signaled by a significant relationship between ASU and the mean indifference point for long delays (*r* = 0.29; *P* = 0.031) and by a correlation between ASU and discounting rate that fell just short of statistical significance (*r* = 0.260; *P* = 0.053).

While these findings suggest only modest correlation effect sizes, and certainly do not justify any claims regarding causality, this now repeatedly observed significant association between mobile technology habits and intertemporal preference is nonetheless intriguing. A tendency to be unwilling to wait for rewards can be a dangerous trait, which has been previously linked to negative outcomes such as drug abuse and gambling addictions (for reviews, see Bickel and Marsch, 2001 and Reynolds, 2006). If heavy use of one’s mobile phone has the power to exacerbate this trait, then there is good reason to be wary of societally increasing usage patterns. If, on the other hand, possessing the natural tendency to seek more immediate gratification drives certain individuals toward heavier phone dependence, broader efforts to monitor habits among those individuals who exhibit a strong orientation toward immediate rewards might be called for, in order to protect against greater vulnerability to the draw of mobile technology devices.

Toward understanding the specific trait characteristics that might motivate heavier smartphone use we also explored potential associations between the smartphone use measures and construct measures for impulse control and reward sensitivity. Although we have previously observed a significant relationship between MTE scores and impulse control using the same measures (Wilmer and Chein, 2016), in the present study this relationship was non-significant (*r* = −0.051; *P* = 0.605) and thus failed to replicate that earlier finding. Instead, the present results indicated a significant positive association between reward sensitivity and MTE (*r* = 0.354; *P* < 0.001), a result consistent with similar correlations reported by Pearson *et al.* (2013) and Sanbonmatsu *et al.* (2013). It should be noted, however, that the absence of a corroborating relationship between reward sensitivity and our objective measure of media use behavior (ASU) warrants some interpretive caution. The inconsistent pattern of association with subjective *vs* objective use measures might signal meaningful differences in the variance that is captured by each approach to measurement.

Indeed, the availability of both a subjective and objective measure of smartphone use offered an important opportunity to assess nuanced aspects of the correspondence between subjective (self-reported) beliefs and actual use patterns. Specifically, we compared participants’ objectively recorded usage of particular social media platforms (Facebook, Twitter, Instagram and Snapchat) in relation to their self-reported claims regarding the amount of time they typically spend on these platforms (as reported on the MTE). As can be seen in Table 3, participants’ categorical self-report estimates corresponded well, if imperfectly, with actually recorded usage times. That is, average actual use corresponded in a generally monotonic fashion with each self-report category and were proximal to the estimated time boundaries (e.g. those reporting 10–20 min of daily Facebook usage actually averaged 24 min of use).

**Table 3:** Actual time spent using Facebook/Twitter/Instagram/Snapchat each day in relation to self-reported estimates of daily usage

	***n***	**Mean**	**Minimum**	**Maximum**	**s.d.**
Facebook					
Rarely ever use	5	5.83	1.14	15.43	5.79
5–10 min	5	13.2	6	30.86	10.3
10–20 min	16	24.23	4.86	66	17.53
20–40 min	5	41.14	16.29	62.57	18.34
40–60 min	5	53.66	17.14	104.57	33.03
Over an hour per day	2	109.71	95.14	124.29	20.61
Total	38	30.95	1.14	124.29	29.53
Twitter					
Rarely ever use	2	6.5	2.71	10.29	5.35
5–10 min	6	10.52	4.29	20.57	6.32
10–20 min	8	16.79	3.14	24	8.59
20–40 min	8	22.61	12.86	33.43	7.33
40–60 min	5	35.31	17.14	66	18.45
Over an hour per day	4	46.29	22.29	99.43	35.88
Total	33	22.82	2.71	99.43	18.59
Instagram					
Rarely ever use	1	2.57	2.57	2.57	-
5–10 min	3	12.19	6.57	17.14	5.38
10–20 min	9	27.63	5.29	89.14	24.39
20–40 min	24	23.94	5	46.29	12.19
40–60 min	8	39.48	6.43	70.29	18.28
Over an hour per day	5	49.37	19.71	125.14	42.94
Total	50	28.5	2.57	125.14	21.7
Snapchat					
Rarely ever use	2	20.14	6.86	33.43	18.79
5–10 min	6	23.14	10.29	42	12.57
10–20 min	12	17.58	4.57	46.29	13.93
20–40 min	13	27.49	10.29	48.86	12.65
40–60 min	9	38.59	5.29	90.86	24.74
Over an hour per day	8	38	7.43	95.14	28.29
Total	50	27.98	4.57	95.14	19.73

All data are shown in minutes of usage, based on the actual battery usage record from the seven days leading up to the laboratory assessment. Variation in the total sample size for each application reflects varying use of these platforms among the sampled population.

## Study 2

The findings from Study 1 support the generalizability of the MTE measure and provide evidence corroborating previously reported associations between technology habits and both DD and reward responsivity. In light of further evidence of a link between frontostriatal white matter connectivity and DD (van den Bos *et al.*, 2014; Hampton *et al.*, 2017), in Study 2 we sought to determine whether the specifically implicated white matter pathways might also be predictive of individual variation in smartphone usage habits. Accordingly, Study 2 combined behavioral and self-report assessments with diffusion imaging of participants’ white matter to explore potential correlations between white matter connectivity, reward processing, intertemporal preference and MTE, with the specific goal of exploring potential dissociations for particular frontostriatal white matter pathways. Specifically, we anticipated that we would replicate the significant associations observed in Study 1 between self-reported MTE and both intertemporal preference and reward sensitivity. Moreover, given the evidence of linkage between reward-related processes and MTE, we anticipated that brain pathways most strongly associated with reward-relevant processing would explain MTE variation.

## Methods

### Participants

Thirty participants were recruited from the Philadelphia area. This sample size was chosen *a priori* based on a power analysis from an earlier study using similar diffusion imaging parameters (Hampton *et al.*, 2017). Participants were all students at either Temple University (a large public and privately funded urban institution) or the University of Pennsylvania (an elite private university). Three participants were excluded because they voluntarily exited the study before completion or failed to follow task instructions. An additional participant was excluded from the sample because of an error recording the diffusion sequence. The final sample size was 26 participants (15 female; age *M* = 21.41 years; s.d. = 2.64). The sample was racially diverse (41.2% self-identified as Caucasian, 23.5% as Asian, 17.6% as African–American and 11.8% as more than one race). All participants were >18 years of age and fluent in written and spoken English.

### Measures

Prior to scanning, participants completed the MTE scale (*α* = 0.76), impulse control (go/no-go; Barratt impulsivity *α* = 0.68) and reward sensitivity (Zuckerman *α* = 0.85; BAS *α* = 0.67) measures and the DD task, all as detailed in Study 1.

### Neuroimaging acquisition

Neuroimaging data were collected for all participants via a 3 T Siemens Magnetom Verio syngo MR B17 scanner (Erlangen, Germany), located within the Temple University Hospital, using a conventional 12-channel phased-array head coil. DWI data were collected using a diffusion-weighted echo-planar imaging sequence covering the whole brain. Imaging parameters were as follows: axial slices, 55; slice thickness, 2.5 mm; repetition time (TR), 9900 ms; echo time (TE), 95 ms; field of view (FOV), 240 mm^2^; *b*-values of 0 and 1000 s/mm^2^, in 64 non-collinear diffusion directions. In addition to diffusion-weighted images, high-resolution anatomical images (T1-weighted 3D MPRAGE) were also acquired using the following parameters: axial slices, 160; slice thickness, 1 mm; TR, 1900 ms; TE, 2.93 ms; inversion time, 900 ms; flip angle, 9^°^; FOV, 256 mm^2^.

### Selection of regions of interest

*A priori* seed regions for tractography were derived based on existing neuroimaging findings pertaining to DD, reward and impulse control. fMRI studies have provided robust evidence that reward-based decision-making is governed by an interconnected circuit of brain regions including the vSTR and vmPFC, as well as areas involved in cognitive control such as the dlPFC (Haber and Knutson, 2010). Masks for the vSTR were taken from the FMRIB Software Library (FSL) Oxford-Imanova Probabilistic Connectivity Striatal Atlas (Tziortzi *et al.*, 2014). As was done in Hampton *et al.* (2017), we subtracted the sensorimotor and executive regions as defined in the atlas, leaving us with only the most ventral (limbic) portion for our analyses. For the dlPFC, we used the cluster masks for Brodmann’s areas 46 and 9 and constructed lateralized masks as defined by the Sallet Dorsal Frontal Connectivity Atlas (Sallet *et al.*, 2013). We defined the vmPFC using a normalized region of interest (ROI) as was originally detailed in Bartra *et al.*, 2013 (positive > negative contrast in Bartra *et al.*’s Figure 3D) creating lateralized ROIs by subtracting a mask of the contralateral hemisphere from each side.

All pre-processing was performed using FSL (Smith *et al.*, 2004). Because we intended to investigate specific white matter pathways, it was necessary to utilize ‘seeded’ tractography, which we did using the vSTR as the primary seed region and the dSTR as a control seed region. The seed in each analysis provided a point from which the white matter that reaches the target ROIs originated. All tractography analyses were performed in the subjects’ native space, and results were output in Montreal Neurological Institute standard space. The FMRIB Diffusion Toolbox toolbox was utilized to conduct probabilistic tractography, using a partial volume model and up to two fiber directions in each voxel. All probabilistic tractography was conducted for each hemisphere, using an exclusion mask to isolate it from the contralateral side. From this method, we acquired the number of ‘streamlines’ (probabilistic connections) that exist between the seed and each target ROI. The number of streamlines was used as an indicator of connectivity strength (van den Bos *et al.*, 2014, 2015; Hampton *et al.*, 2017). To correct for inter-individual variability in the size of each ROI, we divided the number of detected seed-to-target streamlines by the total number of non-zero voxels in the native space seed region and divided the number of target-to-seed streamlines by the number of non-zero voxels in the native space target region. We calculated the means of these two values to produce a composite measure of connectivity between the seed and each target ROI. Example images of the vSTR–vmPFC and vSTR–dlPFC white matter connectivity paths are illustrated in Figure 2B. It should be noted that the indexing of streamlines is based on a mathematical model of the movement of water molecules through space that approximates the trajectory of axonal bundles but does not measure the actual number of axons contained in the bundle nor clarify whether the connective pathways are excitatory, inhibitory, afferent or efferent. The evidence provided through diffusion approaches has, however, been validated by retrograde tracer injection studies in non-human primates (Donahue *et al.*, 2016). Moreover, indices obtained through diffusion imaging are now commonly deployed to pick up on plastic changes in microstructure and myelination that may arise through repeated activity across experience (e.g. learning and habits) and maturation (Sampaio-Baptista and Johansen-Berg, 2017).

## Results and discussion

Data was analyzed for subjects who completed both the behavioral and neuroimaging protocol. The simple bivariate relationship among the behavioral variables in this small neuroimaging sample, summarized in Table 4 (Supplementary Table S2 provides the complete correlation table for all measures in Study 2), was generally consistent with the pattern of findings from the larger cohort in Study 1, though some of the correlations failed to reach significance in this smaller sample. Most notably, the relationship between MTE and intertemporal preference, as indexed by average indifference point, was of a similar direction and magnitude to that observed in Study 1, though it did not approach statistical significance in this smaller neuroimaging sample (*r* = 0.26; *P* = 0.22), nor was there a significant simple relationship between MTE and log(k) (*r* = 0.08; *P* = 0.70). This apparent deviation from the findings of Study 1 could be explained by the reduced power in this smaller sample but was also likely related to the fact that Study 2 participants were drawn from two separate university cohorts with highly disparate demographic profiles. Indeed, the subsample of the Study 2 cohort recruited from Temple University (*N* = 16) revealed a much stronger and significant positive correlation with MTE (with average indifference point *r* = −0.62, *P* = 0.01; with log(k) *r* = 0.50, *P* = 0.048). As such, we included university cohort (group) as a binary variable in subsequent linear regression analyses.

**Table 4:** Bivariate Pearson correlations for measures in Study 2

	**MTE**	**DD**	**IC**	**RS**	**Age**	**vSTR–vmPFC (r)**	**vSTR–vmPFC (l)**	**vSTR–dlPFC (r)**	**vSTR–dlPFC (l)**	**dSTR–dlPFC (r)**	**dSTR–dlPFC (l)**
**MTE**		−0.263	−0.329^†^	0.625^***^	−0.354^†^	0.47^*^	−0.189	0.132	0.109	0.017	−0.037
**DD**			−0.448^*^	0.078	0.464^*^	−0.521^**^	−0.146	−0.571^**^	−0.502^**^	−0.276	−0.437^*^
**Impulse control**				−0.43^*^	−0.239	0.263	0.382^†^	0.338^†^	0.226	−0.066	0.238
**Reward sensitivity**					−0.07	−0.045	−0.186	−0.176	−0.195	−0.135	−0.262
**Age**						−0.355^†^	−0.215	−0.308	−0.267	−0.126	−0.202
**vSTR–vmPFC (r)**							0.128	0.709^***^	0.536^**^	0.3	0.384
**vSTR–vmPFC (l)**								0.284	0.301	0.06	0.305
**vSTR–dlPFC (r)**									0.813^***^	0.491^*^	0.552^**^
**vSTR–dlPFC (l)**										0.516^**^	0.531^**^
**dSTR–dlPFC (r)**											0.847^***^
**dSTR–dlPFC (l)**											

DD indexed by average indifference point. Impulse control signifies composite impulse scores; reward sensitivity signifies composite reward scores. ^†^*P* < 0.10; ^*^P < 0.05; ^**^P < 0.01; ^***^P < 0.001; r means right; l, left.

A series of linear regression models treating our demographic, cognitive and neural measures as potential predictors of MTE served to test the primary hypotheses^i^. Given our interest in dissociating the functions of particular frontostriatal white matter pathways, we first created a vSTR model, which included measures of connectivity from the seed region, the vSTR, to specific, circumscribed ROIs in the frontal lobe—the vmPFC and the dlPFC. As a control, and as a way to probe neural specificity, we also created a dSTR model using the same prefrontal targets but with the dSTR as a seed. Outcomes from these regression models are summarized in Table 5.

**Table 5:** Linear regression models examining the relationship between MTE and demographic, behavioral and neural predictor variables

		*F*	$\beta$	${R}_{adjusted}^2$
		10.39		0.75^***^
Model 1 vSTR	Age		−0.29^*^	
	Group		0.23	
	DD		0.31^*^	
	Impulse control		−0.25	
	Reward sensitivity		0.51^***^	
	vSTR–vmPFC		0.58^***^	
	vSTR–dlPFC		−0.33^*^	
	vSTR–whole brain		−0.03	
		5.49		0.59^**^
Model 2 dSTR	Age		−0.51^**^	
	Group		−0.04	
	DD		0.22	
	Impulse control		−0.30	
	Reward sensitivity		0.66^***^	
	dSTR–vmPFC		0.23	
	dSTR–dlPFC		−0.12	
	dSTR–whole brain		0.14	

vSTR = ventral striatum; dSTR = dorsal striatum; vmPFC = ventromedial prefrontal cortex; dlPFC = dorsolateral prefrontal cortex. β represents standardized beta coefficient. ^*^P < 0.05; ^**^P < 0.01; ^***^P < 0.001.

**Fig. 1:**
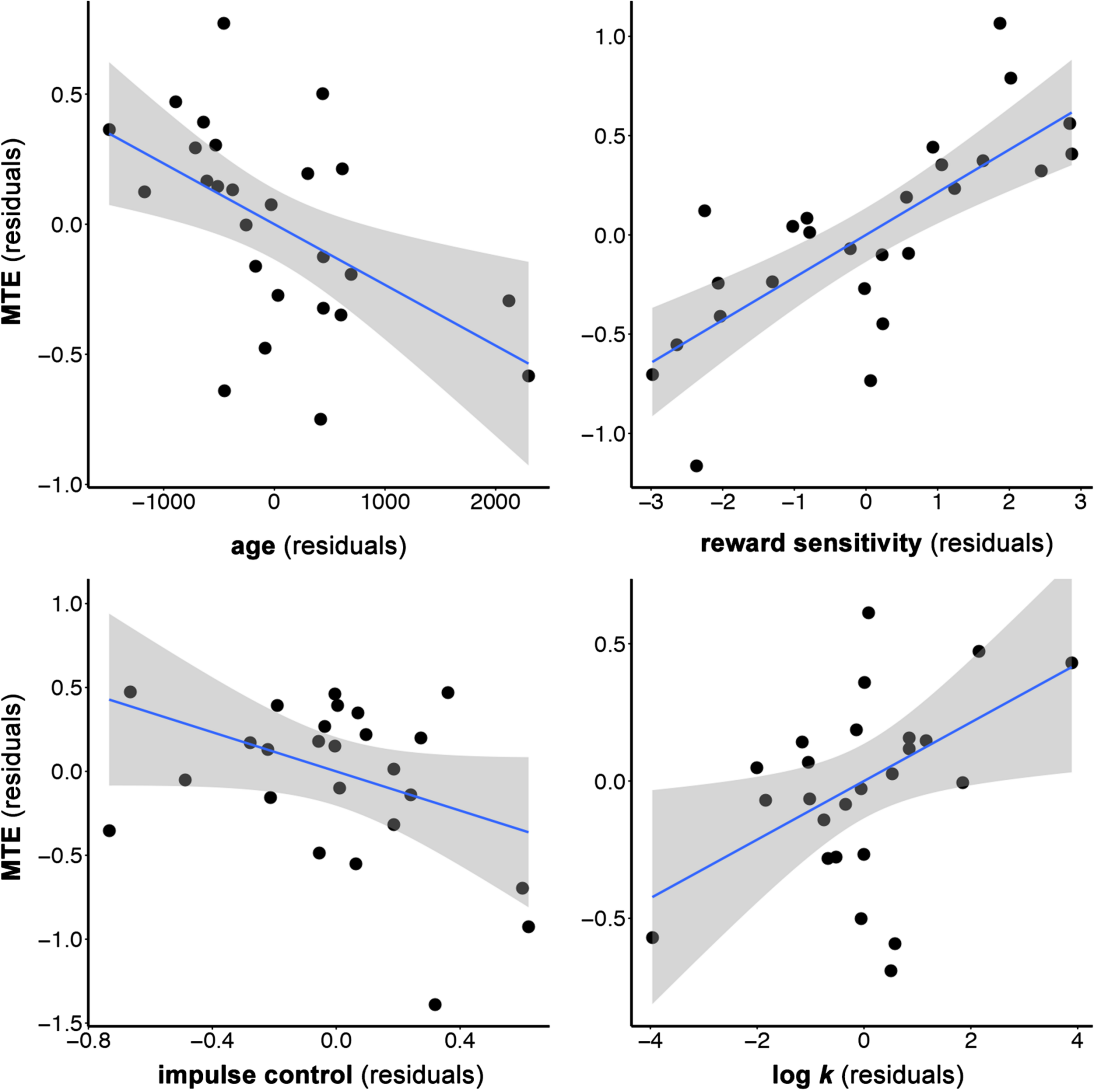
Partial residual plots from the vSTR linear regression model (Model 1 of Table 5), illustrating the relationships between MTE and key variables in Study 2.

**Fig. 2:**
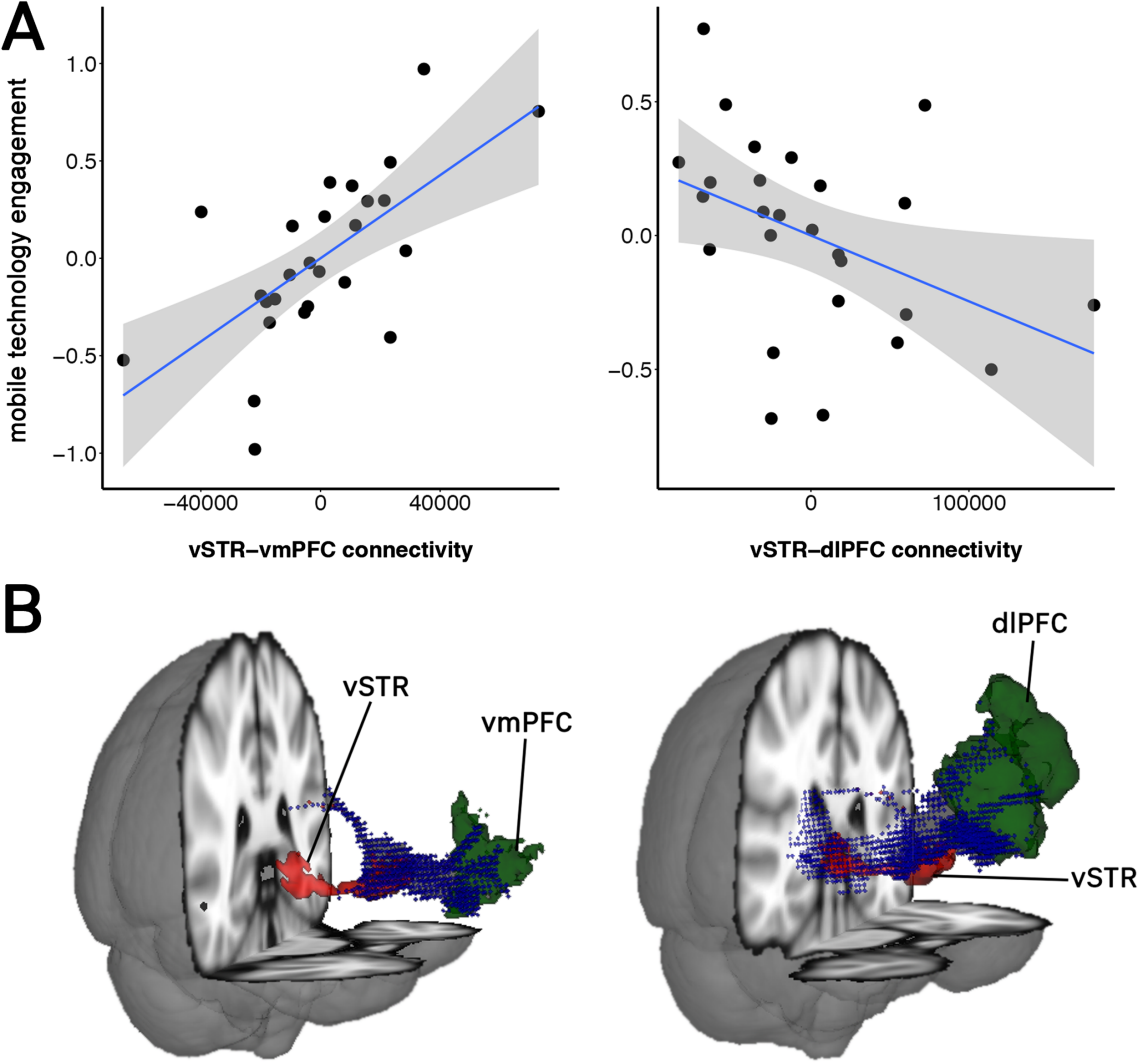
**(A)** Partial residual plots from vSTR linear regression model, illustrating relationship between MTE and white matter connectivity. **(B)** Visualization of white matter computed via probabilistic tractography between the vSTR and the vmPFC, and between the vSTR and dlPFC, for a representative participant.

Overall, the vSTR model was highly significant [Figure 2; *F*(8,17) = 10.39; *P* < 0.001; adjusted *R^2^_adjusted_* = 0.75], such that age [*β* = −0.29 *t*(17) *=* −2.30; *P* < 0.05], intertemporal preference [log(k): *β* = −0.31 *t*(17) *=* 2.14; *P* < 0.05], reward sensitivity [*β* = 0.51 *t*(17) *=* 4.35; *P* < 0.001], right vSTR–vmPFC connectivity [*β* = 0.58 *t*(17) *=* 3.96; *P* < 0.001] and right vSTR–dlPFC connectivity [*β* = −0.33 *t*(17) *=* −2.15; *P* < 0.05] were all associated with MTE. It is also notable that frontostriatal white matter connectivity accounted for a significant amount of variance (∆R^2^ = 0.16) above and beyond that accounted for by the demographic and cognitive predictors alone (as determined by stepwise regression). Of note, the findings from the vSTR model replicates the findings from Study 1 in showing significant associations for both intertemporal preference (log(k)) and reward sensitivity with MTE (Figure 1). In this model, age was also found to be significantly negatively correlated with MTE, while impulse control again exhibited a non-significant association.

As shown in Figure 2, white matter connectivity between the vSTR and target frontal regions exhibited dissociable and opposing relationships with MTE. Specifically, greater vSTR–vmPFC connectivity was associated with ‘higher’ MTE, while, conversely, greater vSTR–dlPFC connectivity was associated with ‘lower’ MTE. Though we must refrain from making causal assumptions in light of the correlational nature of this evidence, the observed pattern of relationship is highly interesting in suggesting that stronger connectivity within a brain pathway typically implicated in the pursuit and processing of rewards (the vSTR–vmPFC pathway) may undergird increased mobile technology use—a finding that is also consistent with the observed associations between MTE and reward responsivity. Meanwhile, stronger connectivity within a pathway typically conceptualized as having a role in the ‘regulation’ of emotional and reward-driven responding (the vSTR–dlPFC pathway) could act to suppress MTE.

To determine whether the particular connectivity pathways we assessed in the vSTR possess a selective relationship with MTE, white matter connectivity from the vSTR to the entire brain (vSTR–whole brain) was also included in the regression model. This connectivity did not significantly predict MTE [*β* = −0.03 *t*(17) *=* −0.29; *P* = 0.77], indicating that the effect was not driven by broader differences in global connectivity to the vSTR. Moreover, while the overall dSTR model was also significant [*F*(8,17) = 5.49; *P* < 0.01], and while dSTR–dlPFC connectivity exhibited an inverse bivariate correlation with DD, none of the dorsal striatal white matter tracts predicted MTE (all *P* > 0.3); only age and reward sensitivity remained significant predictors of MTE in the dSTR model. Therefore, despite some corroborating evidence of an association between dSTR–prefrontal pathways in intertemporal preference (van den Bos *et al.*, 2014), the present data do not provide any evidence that dorsal striatal connectivity is a biological substrate of engagement with mobile technology devices. This result underscores the neuroanatomical specificity of our finding, as only specific ventral, not dorsal, striatum pathways were associated with MTE.

## General discussion

Given the sheer quantity of time that people are now engaged with their mobile devices, it is natural to wonder what effect such habits have on our cognition and on our brain. Toward addressing this question, we drew on a diverse sample of younger adults and collected measures of MTE, phone usage, reward sensitivity, impulse control and DD, in concert with an assessment of brain structural connectivity. By including an objective measure of time spent on one’s phone in the study design, we were also able to show that some self-report assessments of smartphone habits—in this case the MTE—can provide a reasonably apt characterization of the degree to which individuals are invested in their smartphones. While gathering data objectively may yield a more accurate index of phone use patterns, doing so is often less feasible (e.g. based on the type of phone each participant owns and the individual’s willingness to share private usage data) and can be intrusive.

Across two studies, we found corroborative evidence that individuals who exhibit a stronger tendency to discount future rewards also tend to be more engaged with their mobile devices. This is a finding consistent with other recent studies (Atchley *et al.*, 2011; Wilmer and Chein, 2016; Tang *et al.*, 2017). While correlational evidence for this relationship offers no insight into the directionality of the association, it does at least support the plausibility of the contention that heavier mobile technology use might increase impatience and diminish one’s inclination to wait out longer term rewards.

Previous literature has indicated that intertemporal preference as measured by the DD task is a function of two related but separate cognitive traits: reward sensitivity and impulse control; brain imaging evidence demonstrates that these two traits can be isolated to distinct regions of the PFC, with the vmPFC serving as a locus of reward-related processing (O’Doherty *et al.*, 2001; Hampton *et al.*, 2006) and the dlPFC linked to the exercise of control over, and inhibition of, a response (Miller and Cohen, 2001; Hoshi, 2006; Cieslik *et al.*, 2013). Accordingly, we examined whether structural connectivity measures linking the vSTR to these regions could also account for some of the variance in mobile phone usage.

Our interest in this question was driven by prior work showing that impulsivity, as measured by DD, correlates with structural connectivity between the vSTR and portions of the frontal lobe (Peper *et al.*, 2013; van den Bos *et al.*, 2014; Hampton *et al.*, 2017). If increased habitual use of mobile phones is closely linked to impulsive decision-making, aspects of this behavior may rely on the same circuitry. Thus, we aimed to determine if people who used their phones more had different neural connectivity than those who use their phones less.

The present results represent the first evidence that normative individual differences in mobile phone use are indeed reflected in the structural white matter connectivity of our brains. Specifically, we found significant associations between frontostriatal white matter tracts and individual differences in MTE, wherein higher connectivity between the vSTR and the vmPFC—a pathway often implicated in reward processing—was associated with higher scores on the MTE, while higher vSTR connectivity to the dlPFC—an area involved in executive control over behavior—was associated with lower engagement. Notably, neither ‘global’ connectivity of the vSTR, nor connectivity from the dSTR to these same prefrontal areas, predicted MTE (though the left lateralized dSTR–dlPFC pathway did show an association with DD behavior). This pattern of results not only corroborates recent findings indicating that specific frontostriatal connections carry dissociable signals (Hampton *et al.*, 2017) but also that they are differentially predictive of real-world behaviors. Consistent with current views regarding the functions of the vSTR–vmPFC pathway, both studies also provided evidence that the degree to which one engages with mobile technology is tied to individual differences in reward sensitivity and the tendency to pursue near-term rewards at the expense of more beneficial but delayed reward opportunities.

### Limitations

Although we identified specific white matter tracts that were significantly associated with individual variation in MTE, there is little doubt that other pathways would explain additional variance. For instance, we did not examine any cortical–cortical connectivity pathways, which although of interest, are known to be subject to interference of superficial white matter systems for tractographies using diffusion MRI data (Reveley *et al.*, 2015). We urge future researchers to examine the relationship between the other potentially-relevant white matter pathways that may relate to complex real-world decision-making outcomes such as MTE.

In addition, our sample was cross-sectional and therefore does not allow us to adjudicate directionality. Whether high mobile phone use causes changes in white matter, or whether particular white-matter differences predispose certain individuals to engage more with their phones is a matter than cannot be resolved with the present approach. Based on the relatively slow but plastic properties of white matter, it is possible that certain individuals are more prone to engage than others. Engaging in repeated and lengthy use of mobile devices may reinforce these white matter pathways in a bidirectional manner, setting up a positive-feedback loop wherein the pre-existing traits of stronger vSTR–vmPFC connectivity and weaker vSTR–dlPFC connectivity motivate the environmental habit of smartphone engagement, which then produces further plastic change within these pathways.

## Author Contributions

H.W., W.H. and J.C. jointly developed the research concept. H.W. collected data for Study 1 under the guidance of J.C. H.W., W.H. and additional personnel working under J.C., I.O. and T.O. collected data for Study 2. H.W. and W.H. conducted the statistical analyses for Studies 1 and 2, respectively. W.H. conducted diffusion imaging preprocessing, probabilistic tractography and post-processing. H.W., W.H. and J.C. prepared the initial draft of the manuscript, and all authors participated in final editing.

## Funding

This work was supported in part by a National Institute of Health grant [RO1 MH091113 to I.O.].

## Conflict of interest

The content is solely the responsibility of the authors and does not necessarily represent the official views of the National Institute of Mental Health or the National Institutes of Health. The authors declare no competing financial interests.

## Supplementary Material

scan-18-262-File002_nsz024

## Footnotes

^i^ Before building the regression models we first tested possible gender differences for variables of interest and found no significant effects. Accordingly, gender was not included as a variable in the reported regressions. In *post-hoc* regression analyses including gender as an additional variable, we confirmed that gender is not a significant predictor in either overall regression model, and its presence in the model does not change the significance of overall model fit or the significance of other variables of interest in the model.
