# Supplementary material for: Wired to be connected? Links between mobile technology engagement, intertemporal preference and frontostriatal white matter connectivity
Source: Soc Cogn Affect Neurosci. 2019 Apr 25;14(4):367–79. doi: 10.1093/scan/nsz024 (PMC6523422; doi:10.1093/scan/nsz024)
Supplement: scan-18-262-File002_nsz024 [file scan-18-262-file002_nsz024.pdf]

Supplemental Table 1. Bivariate Pearson correlations for all measures in Study 1

		1. MTE	2. MMI	3. MPPUS	4. ASU	5. IP	6. LogK	7. GNG	8. Barratt	9. SS	10. BAS
Smartphone & Social Media Use	**1. MTE**	—	**.39***	**.34****	**.33***	**-.19***	0.12	0.14	0.17	**0.22***	**0.30****
	**2. MMI**		—	0.18†	0.03	-0.04	0.09	**0.22***	0.09	0.10	0.08
	**3. MPPUS**			—	0.06	-0.03	0.04	0.03	**0.21***	0.14	0.17†
	**4. ASU**				—	**-.29***	0.26†	0.04	0.06	-0.08	0.12
Intertemporal Preference	**5. Indifference Point**					—	**0.87*****	0.02	0.14	-.02	0.06
	**6. LogK**						—	0.08	-0.16	.09	0.003
Impulse Control	**7. Go/NoGo False Alarms**							—	0.13	0.11	0.05
	**8. Barratt Impulsivity**								—	**-0.40***	-0.14
Reward Sensitivity	**9. SS**									—	0.17†
	**10. BAS - Reward**										—

Supplemental Table 2. Bivariate Pearson correlations for all measures in Study 2

		1. MTE	2. DD	3. logk	4. GNG	5. Barratt	6. SS	7. BAS	8. vSTR-vmPFC (right)	9. vSTR-vmPFC (left)	10. vSTR-dlPFC (right)	11. vSTR-dlPFC (left)	12. dSTR-vmPFC (right)	13. dSTR-vmPFC (left)	14. dSTR-dlPFC (right)	15. dSTR-dlPFC (left)
Smartphone & Social Media Use	1. Mobile Technology Engagement	—	-0.263	0.078	0.203	0.359†	0.429*	0.693***	0.47*	-0.189	0.132	0.109	-0.092	0.066	0.017	-0.037
Intertemporal Preference	2. Delay Discounting		—	-0.823***	0.56**	0.189	0.12	0.026	-0.521**	-0.146	-0.571**	-0.502**	0.136	-.438*	-0.276	-0.437*
	3. Logk			—	-0.52**	-0.296	-0.173	-0.185	0.332†	0.284	0.467*	0.432*	-0.076	.458*	0.197	0.285
Impulse Control	4. Go/NoGo False Alarms				—	0.321	0.421*	0.33	-0.399*	-0.362†	-0.449*	-0.43*	0.176	-0.077	-0.215	-0.435*
	5. Barratt Impulsivity					—	0.332	0.291	-0.038	-0.284	-0.104	0.075	0.362	-0.192	0.286	0.007
Reward Sensitivity	6. SS						—	0.723***	-0.209	-0.202	-0.286	-0.254	-0.32	0.275	-0.265	-0.416*
	7. BAS - Reward							—	0.093	-0.146	-0.041	-0.084	-0.164	0.025	-0.016	-0.068
Structural Connectivity	8. vSTR-vmPFC (right)								—	0.128	0.709***	0.536**	-0.072	-0.086	0.3	0.384†
	9. vSTR-vmPFC (left)									—	0.284	0.301	-0.362	-0.011	0.06	0.305
	10. vSTR-dlPFC (right)										—	0.813***	-0.081	0.097	0.491*	0.552**
	11. vSTR-dlPFC (left)											—	-0.177	0.353	0.516**	0.531**
	12. dSTR-vmPFC (right)												—	0.009	0.121	-0.085
	13. dSTR-vmPFC (left)													—	-0.041	0.002
	14. dSTR-dlPFC (right)														—	0.847***
	15. dSTR-dlPFC (left)															—
